# Spontaneous Coronary Artery Dissection Involving the Left Main with Extension to Left Anterior Descending Artery and Left Circumflex Artery: Diagnostic and Management Challenges

**DOI:** 10.3390/diagnostics15010061

**Published:** 2024-12-29

**Authors:** Constantin Andrei Rusali, Lucia Cojocaru, Ioana Caterina Lupu, Cezar-Dan Uzea, Maria Lavinia Rusali

**Affiliations:** 1 Department of Cardiology, Constanta County Clinical and Emergency Hospital, Ovidius University of Constanta, 145 Tomis Boulevard, 900591 Constanta, Romania; andrei1678@yahoo.com; 2 Department of Cardiology, Constanta County Clinical and Emergency Hospital, 145 Tomis Boulevard, 900591 Constanta, Romania; uzea.cezar@yahoo.com; 3 Internal Medicine Department, Faculty of Medicine, Ovidius University of Constanta, 145 Tomis Boulevard, 900591 Constanta, Romania; lavinia567@yahoo.com

**Keywords:** case report, spontaneous coronary artery dissection, coronarography, intravascular ultrasound, conservative management

## Abstract

Spontaneous coronary artery dissection is a rare cause of unstable angina, myocardial infarction, and sudden cardiac death, particularly among young women and individuals without conventional atherosclerotic risk factors. We present the case of a 43-year-old woman who had spontaneous coronary artery dissection involving the left main with extension to left anterior descending artery and left circumflex artery. She was ultimately managed medically, with a good outcome. Spontaneous coronary artery dissection is a unique and intricate condition that demands advanced diagnostic techniques and tailored management strategies. Greater awareness and advancements in imaging technologies have enhanced the detection and understanding of spontaneous coronary artery dissection. However, continued research is crucial to resolving outstanding uncertainties and optimizing patient outcomes.

**Figure 1:**
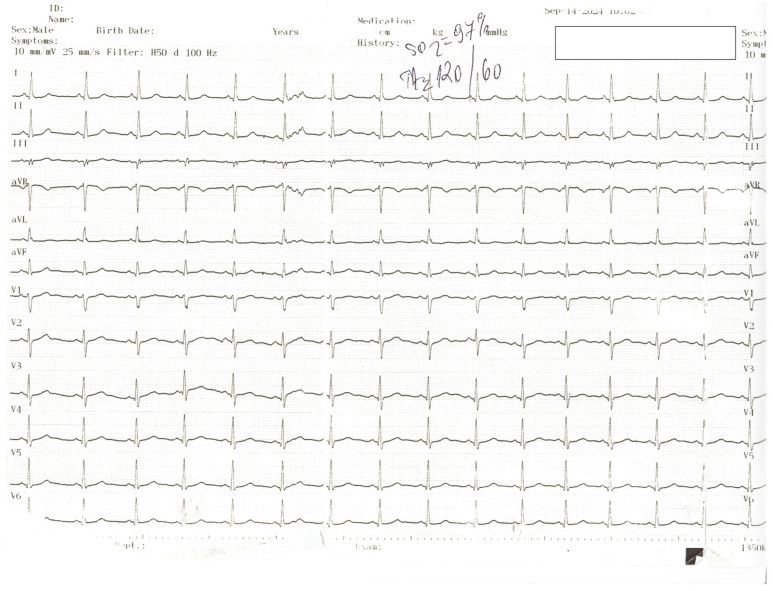
The ECG of a 43-year-old female patient presenting to the Emergency Department (ED) with recurrent retrosternal chest pain at rest, radiating to the back, lasting approximately 10 min, with no significant ST-T changes on the electrocardiogram. The physical exam did not reveal any pathological signs; blood pressure was 120/60 mmHg bilaterally, and heart rate was 60 beats per minute. Echocardiography revealed akinesia of one-third of the anterior, inferior, and lateral walls and septum, with an estimated ejection fraction of 45%. No pericardial effusion was noted, and the aorta had normal dimensions and no intimal flap. Serological results showed normal cardiac necrosis markers. Of note, the patient denied significant medical family history. She was being treated for chronic anxiety.

**Figure 2:**
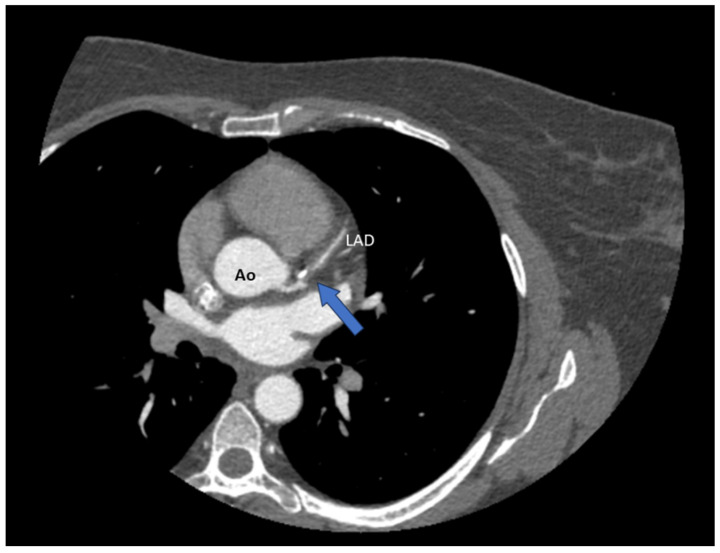
Seeing as the patient had a low suspicion for atherosclerotic disease, we first performed a CT coronary angiography (CTCA), which revealed a significant stenosis of the left anterior descending artery (LAD) (blue arrow); Ao = aorta.

**Figure 3:**
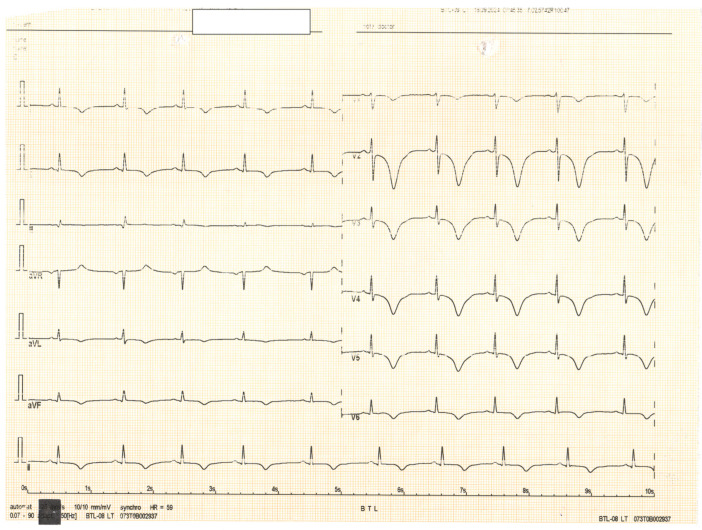
The patient later experienced another episode of resting chest pain. An ECG performed at that time revealed deep, diffuse negative T waves in V1–V6, DI, DII, aVL, and aVF.

**Figure 4:**
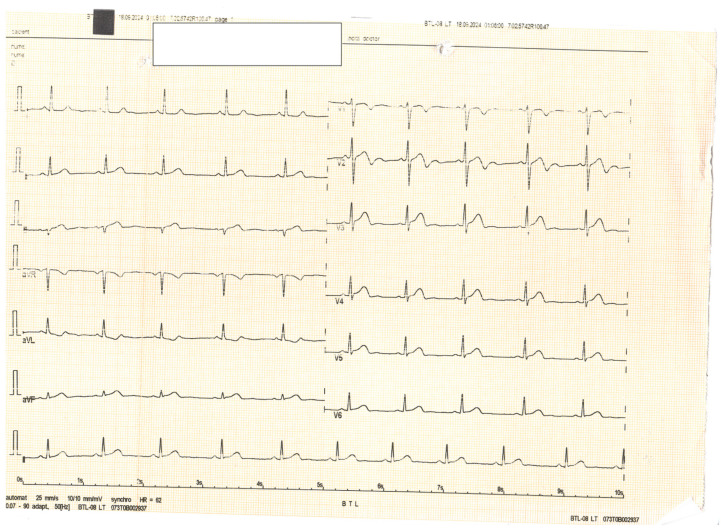
Subsequent ECGs demonstrated the evolution of the ST-T segment, consistent with a Wellens syndrome pattern. Cardiac necrosis markers increased.

**Figure 5:**
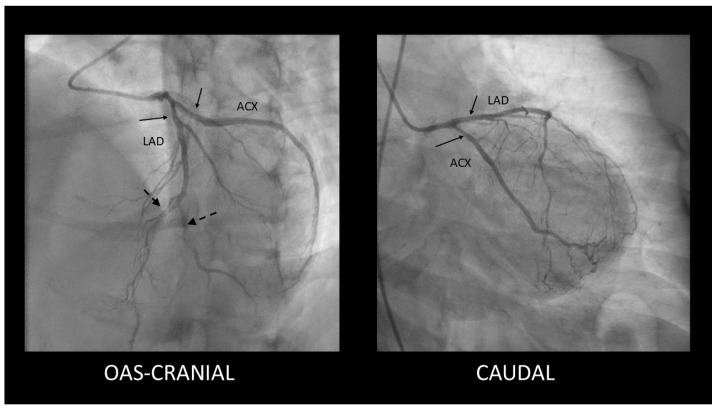
We performed urgent coronarography, which, surprisingly, revealed mild, smooth stenoses in the proximal segments of the LAD and Cx coronary arteries (solid arrows) along with distal stenoses in the mid-LAD and diagonal branch (dashed arrows).

**Figure 6:**
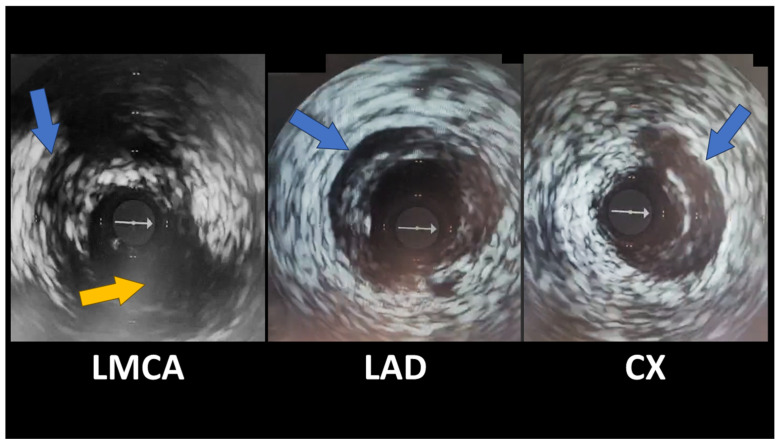
Given the striking discrepancies between the patient’s symptoms, ECG, and CTCA results, we decided to investigate further, and an intravascular ultrasound (IVUS) was performed. It revealed spontaneous coronary artery dissection (SCAD) in the distal left main artery, with extensions in the LAD and proximal circumflex arteries, with hematomas present, classified as a type 2a (Figure 6—spontaneous coronary artery dissections shown by blue arrows; Cx emergence from LMCA—yellow arrow; there is non-significant plaque on LMCA; grey arrow was generated by IVUS and represents a probe marker). The lesions in mid-LAD and the diagonal branch were focal dissections. Given that the hematomas were most likely favored by parenteral anticoagulation, we withdrew this medication, maintaining dual antiplatelet therapy with aspirin and clopidogrel. Conservative management was chosen for this patient because she was hemodynamically stable, angina-free, with normal coronary flow, and without significant atherosclerotic stenoses at the moment of coronarography. We considered that angioplasty with stent implantation had a higher risk due to fragile coronary vessels. The potential benefit of stent implantation arises from sealing the intimal flap. Still, it is overcome by the risk of secondary iatrogenic dissection, guidewire passage into the false lumen, proximal and/or distal false lumen propagation during stent deployment, persistent distal dissection, and major side branch restriction or occlusion by the propagation of hematoma. The patient was discharged in good health with minimal cardiac medication (aspirin, clopidogrel, beta-blocker, ACE inhibitor, and statin) and scheduled for a follow-up coronarography at the 3-month mark (which had not elapsed at the moment of writing this article). We chose to continue dual antiplatelet therapy based on current European Society of Cardiology (ESC) recommendations. According to ESC guidelines, Optical Coherence Tomography (OCT) studies have shown evidence of high-grade stenosis, sometimes with true luminal thrombus associated with SCAD, justifying DAPT in the early phases. Usually, aspirin and clopidogrel are preferred over more potent antiplatelet agents [1]. The true incidence of SCAD remains uncertain, but it is increasingly recognized as a cause of acute coronary syndrome (ACS), particularly in young to middle-aged women [1,2]. There are several known predisposing factors for SCAD: systemic inflammatory conditions, hormonal therapy, fibromuscular dysplasia, postpartum status, multiparity (≥4 births), and connective tissue disorders [3]. Besides these predisposing factors, specific triggers can precede an anginal episode; these include emotional stress, exercise, or hormonal changes (in vitro fertilization [4], hormonal replacement therapy [5], hormonal therapy in transgender individuals [6], oral contraception [7]) [8]. We believe that possible risk factors for SCAD in our patient were gender, age, peri-menopausal status, and emotional stress. CTCA helps assess coronary anatomy and lesions in patients with an uncertain ACS diagnosis, but it can lead to suboptimal results regarding SCAD, i.e., false negatives or misdiagnosis as atherosclerosis, especially in small vessels. SCAD diagnosis on CTCA can be difficult due to its lower spatial and temporal resolution than coronary angiography. The best imaging technique for diagnosing SCAD is IVUS or OCT, which can identify features such as intimal tears, false lumen, intramural hematoma (IMH), and intraluminal thrombi [1]. Management of spontaneous coronary dissection is not completely clarified. Both the European Society of Cardiology (ESC) and the American Heart Association (AHA) have published reports with recommendations. Both reports highlight conservative management as the preferred strategy [1,9]. The ESC states high healing rates over time (73%-100%), and the AHA emphasizes that some cases need to repeat angiography to confirm healing, typically after 35 days. Revascularization may be challenging due to fragile coronary vessel walls, and angioplasty may have worse outcomes compared to atherosclerotic coronary disease. Close monitoring after the index event is recommended by both associations [1,9], with the AHA preferring extended inpatient monitoring for up to 7 days [9]. According to the ESC, 3.3% of conservatively managed patients may require revascularization during follow-up [1]. The AHA states that the first 7 days are critical as patients may experience recurrent MI and need emergency revascularization [9]. In regard to percutaneous coronary intervention (PCI), both reports state that angioplasty is associated with significant complications (false lumen propagation and iatrogenic dissection) and procedural failure (stent restenosis and thrombosis), which may need emergent coronary artery bypass graft (CABG) [1,9]. In general, angioplasty should only be attempted if strictly necessary. Both the ESC and AHA agree that CABG is reserved as a bail-out strategy when PCI fails, especially in cases of ongoing ischemia, infarction, or technical failure (e.g., an inability to wire the true lumen). Left main coronary artery involvement or multiple dissections could require CABG [1,9]. CABG complications are graft failure, which can occur due to competitive flow due to the healing of the native coronary artery. CABG does not prevent recurrent SCAD. When performing CABG, one of the main concerns is anastomosis to the true lumen. The AHA emphasizes a conservative strategy regarding stable patients [9]. Given that SCAD involves bleeding and hematoma formation, the use of traditional acute coronary syndrome drugs such as antiaggregant and anticoagulation is controversial. Aspirin and clopidogrel are the preferred antiplatelet therapy. While the ESC endorses dual antiplatelet therapy (DAPT) [1], the AHA has reservations about this due to a lack of evidence and recommends single antiplatelet therapy (SAPT) in patients not undergoing PCI [9]. Both guidelines raise concerns about anticoagulation due to increased bleeding risk [1,9]. The AHA recommends discontinuing anticoagulation therapy unless it is necessary for other comorbidities [9]. Because SCAD has a non-atherosclerotic pathophysiology, statins are not particularly useful. Statins are recommended if the patient has dyslipidemia or diabetes. Hormonal contraception and hormone replacement therapy carry a potential risk for SCAD, especially since SCAD occurs often in young women, including during pregnancy or postpartum. Evidence in this direction is inconclusive, and no causal link has been established. Ischemia relief can be obtained using traditional antianginal medication (nitrates or calcium channel blockers).

## Data Availability

The data underlying this article will be shared on reasonable request to the corresponding author.
